# Enhancement of Deep Neural Network Recognition on MPSoC with Single Event Upset

**DOI:** 10.3390/mi14122215

**Published:** 2023-12-07

**Authors:** Weitao Yang, Wuqing Song, Yaxin Guo, Yonghong Li, Chaohui He, Longsheng Wu, Bin Wang, Huan Liu, Guang Shi

**Affiliations:** 1 School of Microeletronic, Xidian University, Xi’an 710071, China; 2 School of Nuclear Science & Technology, Xi’an Jiaotong University, Xi’an 710049, China; 3 Dipartimento di Automatica e Informatica, Politecnico di Torino, 10129 Torino, Italy; 4 School of Aerospace Science and Technology, Xidian University, Xi’an 710071, China

**Keywords:** Zynq UltraScale+ MPSoC, single event upset, deep neural network, fault injection, enhancement

## Abstract

This paper introduces a new finding regarding single event upsets (SEUs) in configuration memory, and their potential impact on enhancing the performance of deep neural networks (DNNs) on the multiprocessor system on chip (MPSoC) platform. Traditionally, SEUs are considered to have negative effects on electronic systems or designs, but the current study demonstrates that they can also have positive contributions to the DNN on the MPSoC. The assertion that SEUs can have positive contributions to electronic system design was supported by conducting fault injections through dynamic reconfiguration on DNNs implemented on a 16nm FinFET technology Zynq UltraScale+ MPSoC. The results of the current study were highly significant, indicating that an SEU in configuration memory could result in an impressive 8.72% enhancement in DNN recognition on the MPSoC. One possible cause is that SEU in the configuration memory leads to slight changes in weight or bias values, resulting in improved activation levels of neurons and enhanced final recognition accuracy. This discovery offers a flexible and effective solution for boosting DNN performance on the MPSoC platform.

## 1. Introduction

In recent years, there has been a rapid development in deep neural networks (DNNs), and the implementation of DNN accelerators on Field Programmable Gate Arrays (FPGAs) has gained popularity due to their advantages, such as low power consumption, high integration, and flexibility [1,2,3,4,5,6]. At the same time, machine learning on advanced all-programmable multiprocessor systems on chips (MPSoCs) has also gained traction [7,8,9,10,11,12]. Vendors like AMD/Xilinx have released white papers on DNN implementation with INT4 optimization on Zynq UltraScale+ MPSoC and Zynq-7000 SoC [7].

Numerous studies have explored neural network implementation on advanced MPSoCs using various models and techniques [10,11,12,13,14,15,16,17,18,19,20]. However, these MPSoCs, which are manufactured with scaled technology, such as the 16nm FinFET technology, are susceptible to single event upsets (SEUs) in irradiative environments. It is widely recognized that SEUs can cause data errors or device malfunctions, particularly in aerospace [21,22,23,24]. Furthermore, advanced electronic systems on the ground are also susceptible to SEUs and face potential threats from them [25]. This is why continuous research efforts focus on this topic, with the majority of studies concentrating on evaluating and effectively mitigating the risks associated with SEUs. While the negative impacts of SEUs are well understood, the potential positive contributions of SEUs to design, especially in DNN implementation, have not been thoroughly explored yet.

With the implementation of DNNs in MPSoCs, researchers have also started to investigate the influence of SEUs. However, previous reports primarily focused on the negative impacts of SEUs, such as error results and system halts induced by them [26,27,28,29]. None of these studies have discussed the potential positive contributions, such as reinforcement recognition performance, that SEUs could have in DNN implementations. In the current manuscript, we address this gap and explore the possible positive contributions of SEUs in DNN implementations.

To evaluate the impact of SEUs on DNNs implemented on Zynq UltraScale+ MPSoCs, fault injection (FI) is employed into configuration memory (CRAM) of the device. While irradiation tests are commonly used, fault injection offers greater flexibility and serves as a complementary approach to explore soft errors in the target devices [30,31,32,33]. In particular, the reconfiguration capability of static random-access memory (SRAM) based all-programmable MPSoCs, with the support of Xilinx’s xil_fpga library functions, enables more convenient fault injection through dynamic reconfiguration (DR) via the processor configuration access port (PCAP) [34].

In the current work, we conduct fault injection experiments on DNN implementations to examine the influence of SEU on Zynq UltraScale+ MPSoCs. The general belief is that SEUs have negative impacts on designs, making results less reliable [35]. However, contrary to previous understanding and reported efforts, our results demonstrate that SEUs can also have positive contributions to DNN implementation on Zynq UltraScale+ MPSoCs. Certain SEUs can significantly enhance DNN recognition accuracy, as discussed in subsequent sections.

The structure of the paper is as follows: Section 2 provides an introduction to ZyNet and Zynq UltraScale+ MPSoC. Section 3 introduces the DNN and FI design. Section 4 details the FI implementation, and Section 5 presents the results and analysis. Finally, Section 6 draws conclusions based on our findings.

## 2. Zynet and MPSoC

### 2.1. ZyNet Network

ZyNet, developed by K. Vipin [17,36], is a remarkable open-source DNN framework. It is a Python package specifically designed to facilitate efficient DNN implementation on all programmable MPSoCs. ZyNet also provides support for pre-training or onboard training of networks. One of its notable features is the ability to generate efficient Verilog register transfer level (RTL) code, making it suitable for synthesis and implementation on different FPGA development toolkits.

In this study, ZyNet is utilized to implement the DNN architecture tailored for the Modified National Institute of Standards and Technology (MNIST) dataset. The MNIST dataset is dedicated to recognizing handwritten digits from 0 to 9 and consists of a training set comprising 60,000 28 × 28 pixel grayscale images and a test set of 10,000 images [37].

### 2.2. Tested MPSoC

The device under examination is the Zynq UltraScale+ MPSoC, specifically the AMD/Xilinx XCZU3EG-1SFVA625, known for its high-performance hybrid FPGA platform. The chip incorporates two critical components: The Processing System (PS) with Quad Cortex-A53 and dual Cortex-R5 Cores, and the Programmable Logic (PL) manufactured using 16nm FinFET technology [38].

In [17], ZyNet was introduced and implemented on the AMD/Xilinx Zynq-7000 SoC. It reported a remarkable detection speed of ZyNet on the SoC, achieving four times faster performance compared to the Intel i7 computer. This achievement showcased the high efficiency of DNN on all-programmable SoCs. In the current research, ZyNet is implemented on the 16nm FinFET technology Zynq UltraScale+ MPSoC, and in addition to this, fault injection is performed to explore both positive and negative influences from SEU on DNN implementation, with particular emphasis on the positive aspects.

## 3. DNN and FI Design

### 3.1. Tested DNN

The ZyNet implemented in this study is a 6-layer deep neural network. The network utilizes an 8-bit fixed-point data type, where 4 bits are dedicated to representing the integer portion of the weight values. Each hidden layer in the network employs the sigmoid activation function, and the hardmax layer is utilized to determine the output neuron.

The main objective of this research is to thoroughly evaluate the impact of SEUs in the CRAM on the DNN’s performance within the MPSoC. To achieve a comprehensive assessment of SEU effects on ZyNet, five different sets of hidden layers are designed and trained. Table 1 presents the neuron numbers for each hidden layer in these sets, with “A-30” representing the golden benchmark where each hidden layer (H-i) has 30, 30, 30, 30, and 10 neurons, respectively. For the other sets (i-31 indicates that the corresponding ith hidden layer has an additional neuron), the neuron numbers in the remaining layers are kept the same as those in A-30.

### 3.2. FI Design

**Figure 1:**
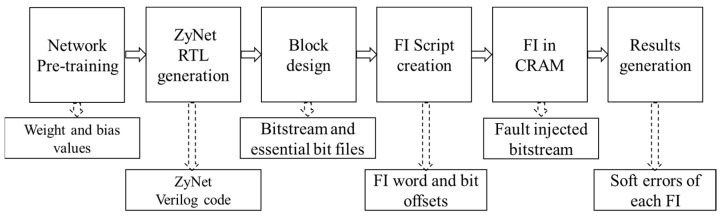
The research framework of each set of DNN.

For each set of DNN, fault injection involves six crucial stages. These stages encompass Network pre-training, ZyNet RTL generation, block design, FI script creation, fault injection, and results observation. Figure 1 illustrates the comprehensive research framework for each set of DNN, depicting the flow of activities and interactions involved in the fault-injection process.

- Network pre-training: For each set of DNN, 50,000 data points are used to train the network, and 10,000 data points are utilized to validate the pre-trained network. The weight and bias values generated during this stage are then used for ZyNet RTL generation;
- ZyNet RTL generation: Verilog codes for the ZyNet RTL module are generated. Subsequently, the ZyNet RTL module is integrated into the block design in Vivado;
- Block design: The Vivado 2019.2 is used as the design toolkits. The generated ZyNet RTL module is added to the block design in Vivado. The direct memory access (DMA) IP is connected to the ZyNet module with the read channel enabled. Additionally, the necessary bits are activated in the constraints. After synthesis and implementation, the bitstream and essential bit files, including essential bit data (EBD) and essential bit configuration (EBC) files, can be obtained [39,40];
- FI Script creation: The FI script is created by extracting a total of 50,000 intended injected bits from the EBD file for each DNN. In the EBD file, the ‘1′ bits represent the essential bits. The FI script includes information about the location of the targeted injection word and bit offsets;
- FI on CRAM: Before loading the bitstream into CRAM from DDR to achieve functionality, a fault is injected into CRAM using the ‘XOR’ operation to flip the targeted bit. Then, the fault-injected bitstream is loaded into CRAM through dynamic reconfiguration over the PCAP interface;
- Results generation: After loading the fault-injected bitstream, the software program is executed, and the results are promptly generated and updated in the terminal. Finally, soft errors resulting from each fault injection are observed.

More details about the fault-injection process are presented Section 4.

### 3.3. DNN Verification

Before conducting the fault-injection process, it is essential to verify the design performance of each DNN on the Zynq UltraScale+ MPSoC. In this study, both software and hardware implementations are performed for verification purposes. The software implementation involves training each DNN for 30 epochs using Python to examine accuracy. On the other hand, the hardware implementation directly evaluates the DNN performance on the Zynq UltraScale+ MPSoC. This involves loading bitstream into CRAM without any fault injection. The purpose of this step is to ensure that the DNN performs as expected on the hardware platform.

The verification results for both software and hardware implementations of each DNN are presented in Table 2. Additionally, the resource utilization of each DNN on the Zynq UltraScale+ MPSoC is summarized in Table 3. The recognition accuracies for both software and hardware implementations are found to be quite close, indicating that the ZyNet platform is effectively implemented on the Zynq UltraScale+ MPSoC. Furthermore, it is observed that the DNN accuracy of the hardware implementation gradually increases as the number of neurons added gets closer to the output layer on the Zynq UltraScale+ MPSoC. This finding highlights the potential benefits of optimizing the DNN architecture to achieve improved performance on the Zynq UltraScale+ MPSoC.

## 4. FI Implementation

**Figure 2:**
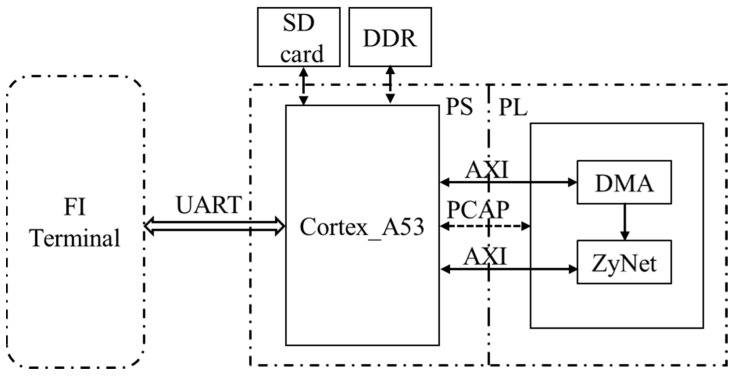
The FI system layout of each DNN.

**Figure 3:**
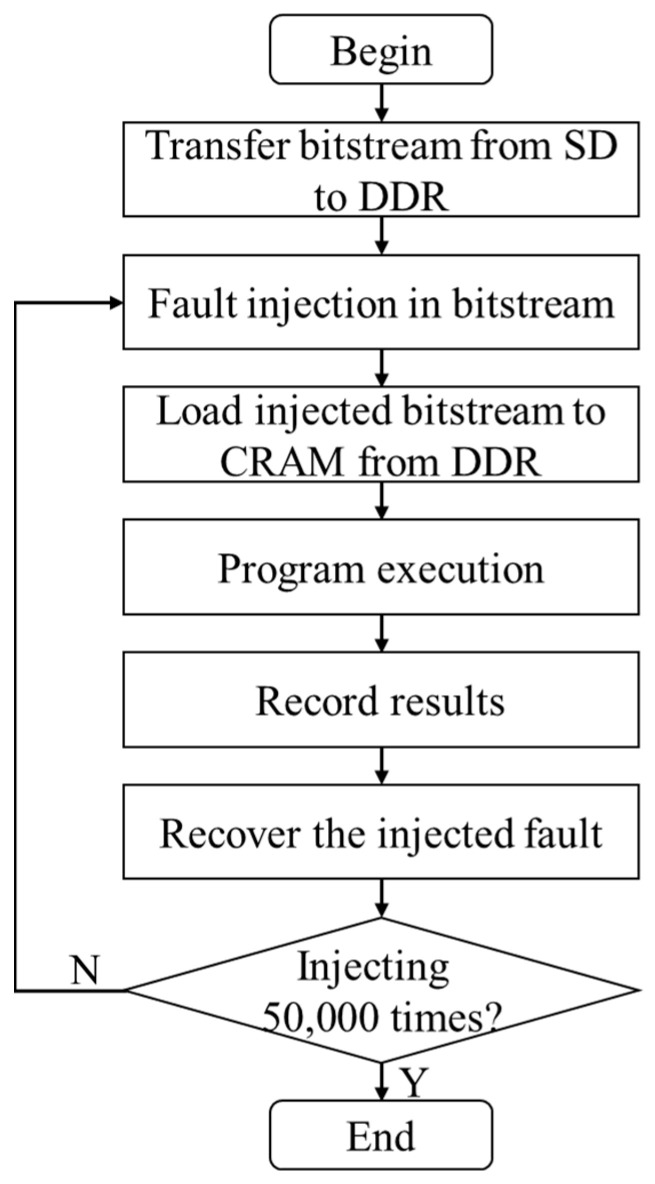
Workflow of FI injection software of each DNN.

In this section, further details are provided on the process of fault injection for each DNN. The hardware block designs for the DNNs, and the software programs where fault injection is executed, are implemented using Vivado 2019.2 and Vitis 2019.2, respectively. Figure 2 provides an illustration of the layout of the FI system, while Figure 3 depicts the workflow of the software program during fault injection. Although there are differences in the layers’ neurons and corresponding bitstreams for each DNN, the overall layout and workflow remain consistent.

In Figure 2, the secure digital (SD) card and double data rate (DDR) synchronous dynamic random-access memory serve as storage for the bitstream, and the PCAP interface is responsible for loading the bitstream into CRAM. The FI terminal allows for setting serial number for the injection to be launched and displays execution messages by communicating with the device over universal asynchronous receiver transmitter (UART). As shown in Figure 3, the FI process occurs before loading the bitstream from DDR to CRAM. Once the program is executed and the results are recorded (examining 10,000 test data) for each injected fault, the injected fault is subsequently recovered. The FI process for each DNN is completed when all 50,000 created FI injection locations have been checked.

As stated, the xil_fpga library functions and dynamic reconfiguration enables more flexible loading of the injected bitstreams into CRAM through the PCAP interface. For example, the XFpga_PL_BitStream_Load() function can repeatedly load the injected bitstream into CRAM, ensuring efficient reconfiguration. Additionally, the DR significantly improves the efficiency of the fault injection, particularly over the PCAP interface, without the need for additional hardware resources or software settings. To save operation time, only the numbers of misidentification (NOM) among the 10,000 test data are output for each fault injection.

Following the same process, faults are injected one by one into the five DNNs according to the sequence presented in Table 1, Table 2 and Table 3. This systematic approach allows for a comprehensive evaluation of the impact of faults on each DNN. It can provide valuable insights into potential vulnerabilities and performance variations in the presence of SEUs.

## 5. Results and Analysis

### 5.1. Detected Soft Errors

The five DNNs are examined one by one, with each DNN subjected to 50,000 injected faults. The observation of these fault injection reveals four distinct types of outcomes: Normal, recognition accuracy varied (RAV), system halt (SH), and DMA initialization failed (DIF). The latter three types are initially classified as errors and become the focus of analysis for each DNN. Although the numbers of errors may vary, the detected error types remain consistent across all DNNs.

The four types of fault injection results are defined as follows:

- Normal: The injected fault has no influence on the identification accuracy, and the NOMs remain unchanged;
- Recognition Accuracy Varied (RAV): In this case, the NOMs among the 10,000 test data differ from normal fault injection results. No further operations are required, and the fault-injection process can continue;
- System Halt (SH): If the program execution is halted, and the UART stops outputting messages, new fault injections cannot proceed. A software reset is necessary;
- DMA Initialization Failed (DIF): In this scenario, the DMA initialization fails, preventing test data being moved to the networks for processing. A software reset is required in this case as well.

Table 4 lists the NOMs for each implemented DNN on the Zynq UltraScale+ MPSoC under the condition of no faults. The NOMs are recorded as 445, 432, 413, 396, and 384, for the respective DNNs. To quantify the impact of fault injections on each DNN’s performance and identify potential vulnerabilities resulting from SEUs, a comprehensive analysis is conducted. For each RAV error, if the NOMs are different from the values in Table 4, they are counted once. For SH and DIF errors, their occurrences are counted directly.

The initial occurrences of errors for each DNN are summarized in Table 5. Among the 50,000 faults, RAV is the most prevalent error type observed for each DNN. Additionally, the number of SH errors is significantly higher than that of DIF errors by an order of magnitude.

### 5.2. Results Analysis

The RAV and SH errors are caused by faults injected into the DNN implementation, specifically targeting essential bits. On the other hand, the DIF errors are induced by faults injected into the DMA IP related essential bits. To measure the error rate of fault injection, the soft error sensitivity (SES) is a critical metric calculated using Equation (1) [41].

SES = N_e_/N_i_ (1)

where SES is the soft error sensitivity, N_e_ is the number of detected errors, and N_i_ is the number of injected faults.

**Figure 4:**
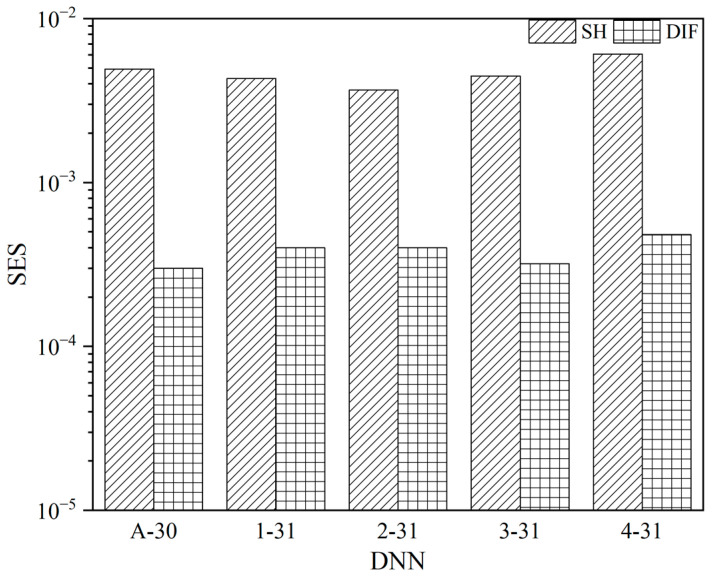
SES values of SH and DIF of each DNN.

In Figure 4, the SES values for SH and DIF errors are calculated and plotted. The maximum and minimum SH SES values are 6.08 × 10^−2^ and 3.66 × 10^−2^, obtained for the 4-31 and 2-31 networks, respectively. Similarly, the maximum and minimum DIF SES values are 4.80 × 10^−3^ and 3.00 × 10^−3^, obtained for the 4-31 and A-30 networks, respectively.

Regarding RAV errors, a noteworthy phenomenon draws attention—the recognition accuracy enhancement (RAE) phenomenon. This phenomenon indicates that in certain fault-injected networks, the number of misidentifications is smaller than that of the normal network. For instance, in the 4-31 network, without fault injection, the NOM is 384. However, in some fault injection cases, this number becomes smaller than 384, resulting in RAE. Conversely, in some cases, the number becomes larger than 384, leading to recognition accuracy degradation (RAD). RAD represents a negative impact introduced by SEU in CRAM and requires measures to be addressed in the CRAM.

In Table 5, the RAE and RAD are counted together as part of RAV. However, in Table 6, the numbers of them are presented separately. It is surprising that although the numbers of RAE are smaller than those of RAD, the percentages of RAE reach 24.1% to 33.2% in all RAV cases, highlighting the significant presence of the recognition accuracy enhancement phenomenon.

### 5.3. RAE of DNN

Until now, the majority of research has primarily focused on the threats and adverse effects caused by SEU in CRAM, with little attention given to potential positive contributions. In most circuit designs within FPGAs, SEU in CRAM typically leads to unexpected negative outcomes. Nevertheless, the presence of the RAE phenomenon in DNN implementations on MPSoCs is both intriguing and reasonable.

#### 5.3.1. SEU induced RAE

**Figure 5:**
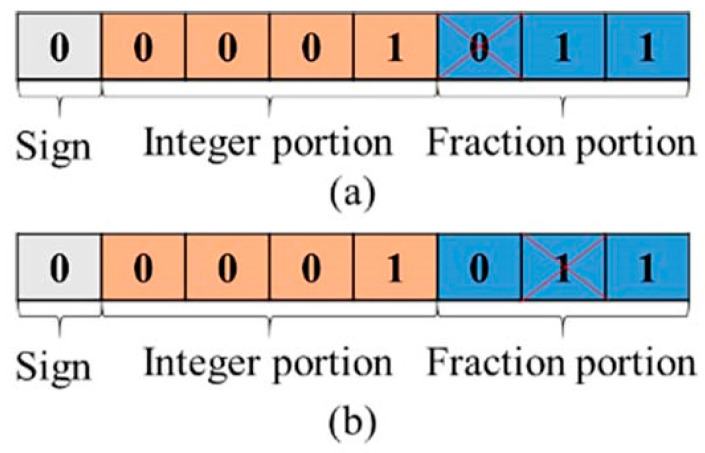
An example of SEU in fraction portion of a weight value: (**a**) SEU in the first fraction bit and (**b**) SEU in the second fraction bit.

In this study, each DNN implementation relies on pre-trained weight and bias values, which are then mapped into LUTs and FFs, and others in the PL part. The weight values, for instance, are represented as 8-bit fixed-point data types. Figure 5 illustrates an example of an SEU affecting the first and second fraction bits of a weight value in (a) and (b), respectively. If this SEU introduces a slight change in the proportion to determine the final outcomes, leading to an increased probability of producing a correct result and minimizing the possibility of misjudgment. This can be considered an enhancement by SEU. Alternatively, the SEU changes the weight or bias of an individual hidden layer in the DNN, and results in a better activation level of neurons. Consequently, it enhances the final recognition accuracy, the RAE phenomenon emerges.

We speculate that the potential for SEU to enhance design performance may not be limited to DNNs implemented on MPSoC. It could also improve the speed, accuracy, and energy efficiency of non-quantitative computation. These call for further in-depth exploration in future efforts.

**Figure 6:**
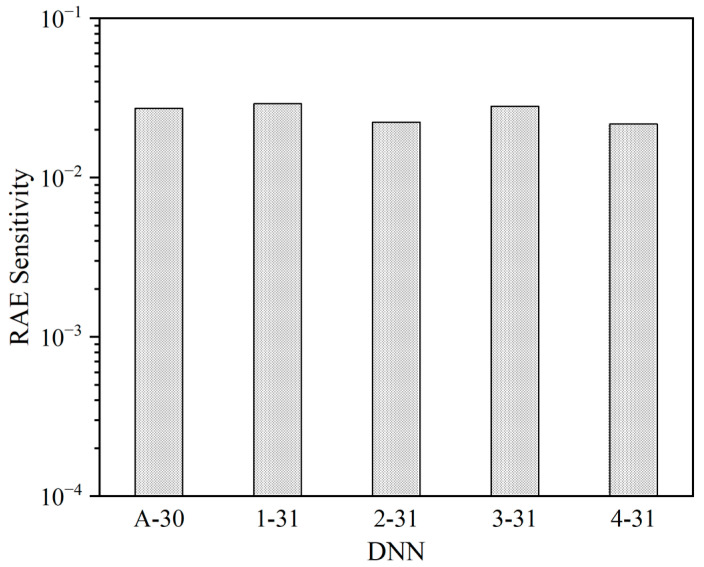
RAE sensitivity of each DNN.

After conducting 50,000 fault injections in each DNN, various numbers of RAEs are detected, as shown in Table 6. The RAE sensitivities are depicted in Figure 6, representing the proportion of RAE occurrences out of the total number of injected faults (i.e., 50,000 faults). The RAE sensitivity values range from 0.022 to 0.029. Importantly, these RAE sensitivities are calculated based on 50,000 fault injections for each DNN. It is reasonable to speculate that as the number of injected bit increases, the significance of RAEs will also increase.

Table 7 presents the essential bit lengths for each DNN. By using the RAE sensitivity values from Figure 6, it is predicted that the maximum number of RAE occurrences will be approximately 148,342 in the 1-31 network. Additionally, the predicted numbers of RAE occurrences for each DNN are summarized in Table 7. These predictions provide valuable insights into the potential impact of RAE phenomena as more faults are injected, guiding future research and mitigation strategies.

#### 5.3.2. Optimal RAE

Moreover, we have identified the optimal recognition accuracy enhancement (ORAE) fault-injection locations in the bitstream of each DNN. The ORAE fault injection refers to the injection that results in the least NOM for each DNN. The NOMs obtained from the ORAE fault-injection locations are presented in Table 8 for each corresponding DNN. These results highlight the extract locations in the bitstream where fault injections can lead to the most substantial improvements in recognition accuracy for each DNN.

As shown in Table 8, the maximum improvement in recognition accuracy reaches 8.72% for the 2-31 network. These results provide a promising solution for enhancing DNN performance on the MPSoC, which will be further discussed in Section 5.4.

**Figure 7:**
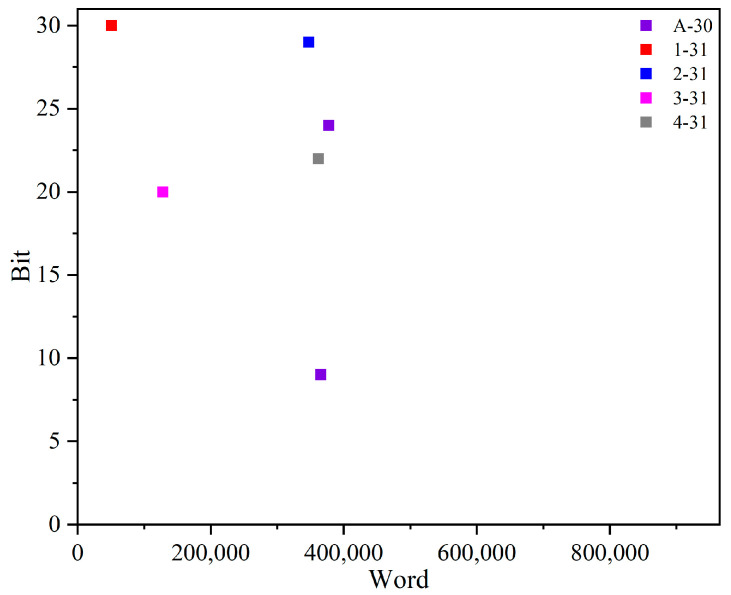
FI locations at ORAE for each DNN.

In addition, the fault-injection technique relies on dynamic reconfiguration, which enables accurate identification of the injected word and bit offsets for each injection. Consequently, the precise locations of ORAE fault injections are easily determined. Figure 7 visually depicts the ORAE fault-injection locations for each DNN. Interestingly, for the A-30 network, two locations produce the same NOM, indicating that there can be multiple fault-injection locations that effectively optimize DNN performance in the MPSoC. This discovery highlights the potential for fine-tuning DNN performance through strategic fault injections in various locations.

### 5.4. Solution for Enhancing DNN Performance

Traditionally, substantial efforts have been dedicated to enhancing DNN performance through the development of complex designs or algorithms aimed at reducing misidentifications [42,43]. However, in the context of DNN implementation on all programmable MPSoCs, we propose a convenient and efficient solution that does not necessitate training a new network. Instead, we achieve performance improvement through direct fault injection, avoiding the need for intricate designs or algorithms. This approach not only enhances DNN performance but also saves on development costs and time.

For illustration, Table 9 presents the design costs for different cases. It becomes evident that achieving similar recognition accuracy would require training additional epochs or adding more neurons. These alternatives incur extra time or resource costs, respectively, whereas our fault injection method offers a simpler, more cost-effective solution for optimizing DNN performance on the MPSoC platform.

To optimize the performance of an implemented DNN on an advanced MPSoC by SEUs, a systematic approach is followed. Firstly, fault injection is performed on CRAM to observe its impact on performance. Secondly, the injection locations that lead to a reduction in NOMs are identified. Thirdly, the optimal injection location(s) are selected. Finally, the bitstream containing the ORAE fault injection is loaded into CRAM, and the program is executed on the MPSoC to improve DNN performance.

Contrary to the common opinions that visible effects of an energetic particle striking CRAM are harmful, this study proves otherwise. In fact, it even demonstrates an 8.72% performance enhancement through fault injection. Although the examined DNN in this study may not be the most complex, the discoveries and proposed solutions are applicable to other intricate designs implemented on advanced SRAM-based fully programmable MPSoCs. The findings highlight the potential benefits of exploring SEU-induced positive contributions for optimizing deep learning systems on advanced MPSoC platforms.

## 6. Conclusions

Fault injections are performed in configuration memory of five sets of deep neural networks implemented on the Zynq Ultrascale+ MPSoC. The fault-injection process emulates single event upset in the device and is based on dynamic reconfiguration over the PCAP interface in the MPSoC. Finally, three types of soft errors are observed, including recognition accuracy varied, system halt, and DMA initialization failed. A significant finding of the research is about 24.1% to 33.2% in all recognition accuracy varied soft errors, enhancing DNN performance based on fault injection results. This means that the identification accuracy of the networks increased compared to when no single event upset occurred. Depending on fault injection, the current research confirms that single event upset can also introduce positive effect on designs, such as DNN implemented on the MPSoC. Additionally, optimal fault-injection locations are identified for each DNN, resulting in the least numbers of misidentifications for each case. Compared to a deep neural network implemented on the MPSoC without upset in some essential bits of configuration memory, the maximum performance enhancement in a fault injected DNN reaches 8.72%. Finally, a solution is proposed to enhance deep neural network performance implemented on SRAM-based full programmable MPSoCs. This solution relies on fault injection to extract the optimal fault injection location and improve overall network performance.

## Figures and Tables

**Table 1:** Numbers of neurons of hidden layers in each set DNN.

Network	H-1	H-2	H-3	H-4	H-5
A-30	30	30	30	30	10
1-31	31	30	30	30	10
2-31	30	31	30	30	10
3-31	30	30	31	30	10
4-31	30	30	30	31	10

**Table 2:** Software and hardware verification results of each DNN.

Network	Recognition Accuracy	
	Software Implementation	Hardware Implementation
A-30	96.21%	95.55%
1-31	96.07%	95.68%
2-31	95.69%	95.87%
3-31	96.14%	96.04%
4-31	96.35%	96.16%

**Table 3:** Resource utilization of each DNN.

Network	Look Up Table (LUT)	LUTRAM	Flip Flop (FF)	Block RAM (BRAM)	BUFG
A-30	16,576 (23.49%)	257 (0.89%)	9995 (7.08%)	42.50 (19.68%)	1 (0.51%)
1-31	16,223 (22.99%)	257 (0.89%)	9998 (7.08%)	45.50 (21.06%)	1 (0.51%)
2-31	15,738 (22.30%)	257 (0.89%)	9957 (7.06%)	47.50 (21.99%)	1 (0.51%)
3-31	16,075 (22.78%)	257 (0.89%)	9975 (7.07%)	45 (20.83%)	1 (0.51%)
4-31	16,475 (23.35%)	257 (0.89%)	10,025 (7.10%)	43.50 (20.14%)	1 (0.51%)

After verification, fault injection can be executed on different DNNs.

**Table 4:** Numbers of misidentification for each DNN.

Network	Number of Misidentification
A-30	445
1-31	432
2-31	413
3-31	396
4-31	384

**Table 5:** Error occurrence numbers of each DNN.

Network	Total	RAV	SH	DIF
A-30	5500	5239	246	15
1-31	4620	4385	215	20
2-31	3971	3768	183	20
3-31	4768	4529	223	16
4-31	4830	4502	304	24

**Table 6:** RAE and RAD numbers of each DNN’s FI.

Network	RAE Number	RAD Number
A-30	1359	3880
1-31	1455	2930
2-31	1114	2654
3-31	1402	3127
4-31	1084	3418

**Table 7:** Essential bit lengths and predicted number of RAE of each DNN.

Network	Essential Bit	Predicted RAE Number
A-30	5,084,661	138,201
1-31	5,097,678	148,342
2-31	4,949,596	110,277
3-31	4,983,812	139,746
4-31	5,093,162	110,419

**Table 8:** Numbers of NOM at ORAE in each set DNN.

Network	NOM	NOM at ORAE	Enhancement
A-30	445	419	5.84%
1-31	432	399	7.64%
2-31	413	377	8.72%
3-31	396	380	4.04%
4-31	384	363	5.47%

**Table 9:** Effort in design recognition accuracy enhancement.

Network	Measurement	Enhancement	Extra Cost
2-31	Flip 1 bit at OREA	8.72%	/
2-31-I	Increase the training epoch from 30 to 60	8.02%	Training time
2-31-II	Add 1 neutron in H-1, H-3, H-4	8.10%	Hardware resource

(2-31-I: training epoch is 60, 2-31-II: the neurons of each hidden layer are 31, 31, 31, 31, 10).

## Data Availability

Data are contained within the article.
